# Efficiency of conventional and nanoparticle oxytetracycline in treatment of clinical endometritis in postpartum dairy cows

**DOI:** 10.1007/s11250-023-03536-0

**Published:** 2023-03-17

**Authors:** Rezk S. Ghallab, Dina R. S. Gad El-Karim, Abdel-Hasseb Fayed, Amr M. A. Rashad

**Affiliations:** 1 Theirogynology Department, Faculty of Veterinary Medicine, Matrouh University, Mersa Matrouh, Egypt; 2 Department of Pathology and Clinical Pathology, Faculty of Veterinary Medicine, Alexandria University, Alexandria, Egypt; 3 Department of physiology, Faculty of Veterinary Medicine, Alexandria University, Alexandria, Egypt; 4 Animal and Fish Production Department, Faculty of Agriculture (El-Shatby), Alexandria University, Alexandria, 22545 Egypt

**Keywords:** Clinical endometritis, Oxytetracycline, Nano, Cytokines, Dairy cows

## Abstract

The incidence of clinical endometritis in dairy cows postpartum is one of the important reasons for financial losses in the dairy industry. The costs of treatment, milk losses, infertility, repeated breeding, and high annual culling rate of dairy cows present immediate losses in case of treatment failure. The commonly used therapeutic methods for clinical endometritis have not been successful nor have given definitive solutions to overcome the complications of the disease in dairy cows. Therefore, it was necessary to propose an innovative treatment program to overcome the reasons for the failure and lack of effectiveness of the treatment of clinical endometritis. This was tackled in the current study; oxytetracycline with different concentrations, oxytetracycline 5% (OTCC5%), oxytetracycline 20% (OTCC20%), and oxytetracycline 20% nanoparticles (OTC-NPs) were used for the treatment of clinical endometritis. Diagnosis of clinical endometritis was based on the assessment of high serum concentration of pro-inflammatory cytokines, acute phase protein, increased endometrium thickness, and intrauterine discharges with different degrees of echogenicity monitored by ultrasonography. Application of OTC-NPs revealed a decrease in serum concentration of pro-inflammatory cytokines (IL-1, IL-6, and TNF-α) and acute phase proteins compared to OTCC20% and OTCC5% groups. The improvement achieved by OTC-NPs may be attributed to the reduction of OTC particles into nano size which facilitates its tissue bioavailability, dispersion, penetration power to deeper tissues, and its more broad-spectrum activities. These activities were clearly apparent after the evacuation of uterine contents using a single dose of PGF2α. The OTC-NPs revealed a reduction in serum concentration of cytokines compared to OTCC20% and OTCC5% groups arranged as follows: 10.11, 25.45, 35.56 for IL-1; 99, 300, 319 for IL-6; 1.01, 4.40, 8.06 for CRP; and 46, 183, 266 for TNF-α. Furthermore, an increase in serum concentration of albumin (3.34) was obtained by OTC-NPs compared to OTCC5% (1.70). This improvement can be taken as evidence of liver resumption functions and inflammatory reactions. On the other side, globulin concentration recorded an increase like albumin and total proteins in OTC-NPs compared to others. A reduction in the endometrium thickness in OTC-NPs with the disappearance of intrauterine discharges was monitored by ultrasonography. This confirmed the subsiding of clinical endometritis in OTC-NPs group. Moreover, a significant improvement in conception and pregnancy rate in OTC-NPs compared to other groups were observed.

**Supplementary Information:**

The online version contains supplementary material available at 10.1007/s11250-023-03536-0.

## Introduction

Postpartum endometritis is one of the major dangerous disorders affecting the reproductive performance of dairy cattle (LeBlanc et al. 2002). It remains a serious economic problem for dairy industry all over the world due to the large financial losses caused by the high rate of artificial insemination failure and the necessity for culling (LeBlanc 2008) or diminishing the profitability of dairy herds (Overton and Fetrow 2008). A strong relationship was reported by Pande et al. (2013) between the resumption of postpartum ovarian cyclicity and cytological endometritis associated with low levels of progesterone. Clinical signs of postpartum endometritis range from temporary and self-limiting signs to chronic or future fertility-threatening ones. Moreover, the treatment of endometritis generally involves hormonal and antibiotic therapy separately or in combination. However, the successful resolution of endometritis mainly relies on the used diagnostic aids which help selection of appropriate therapy (Gustafsson 1984). One of these diagnostics aids are cytokines and acute phase proteins (APPs). In addition, clinical endometritis can be diagnosed by the accumulation of mucopurulent and purulent discharges in the postpartum period (Abdel-Latif et al. 2016). Furthermore, cytokines include a wide category of small proteins (~5–20 kDa) that act as an important cell signaling secreted by immune cells like macrophages, monocytes, and other immune cells, which produce cytokines, chemokines, interferons, interleukins, lymphokines, and tumor necrosis factor and affect the behavior of the secreting cells (Liu et al. 2007). It was evident that the development of bovine endometritis involves very complex signaling processes including the detection of bacterial components by innate immune cells via toll-like receptors; the production of the tumor necrosis factor-alpha (TNF-α) and other pro-inflammatory cytokines, e.g., interleukins (IL); and the mobilization of neutrophils followed by the phagocytosis of invading pathogens within the uterine lumen (Sheldon et al. 2009 and Turner et al. 2012). The mechanism of action of the pro-inflammatory cytokines (e.g., TNF-α, IL-1β, and IL-6) stimulates neutrophil and monocyte diapedesis and chemoattraction and promotes increased phagocytosis (Singh et al. 2008) besides its potent stimulation for the production of APPs, such as haptoglobin, acid glycoprotein, and ceruloplasmin or serum amyloid A (Tóthová et al. 2008). Their task, however, is to assist in the elimination of infection by phagocytosis. Numerous studies proved that the infusion of inflammatory cytokines in uterine tissue was found to be related to clinical or subclinical endometritis due to infection by *Escherichia coli* (*E. coli*) and *Trueperella pyogenes* which are the most common challenges for occurrence of bovine endometritis (Gautam et al. 2010); they can resist antimicrobial efficacy and limit its action (Suojala et al. 2013). Moreover, the continuous presence of broad-spectrum β-lactamase enhances *E. coli* and other species of bacteria to lower the efficiency of antibiotics (Hasman et al. 2005). Indeed, continuous uses of antibiotics and genetic variation of bacteria participate in the occurrence of bacterial resistance and failure of antibiotics to treat endometritis (Aslam et al. 2018). Administration of PGF2α and its analogues have been incorporated into treatment protocols of endometritis; enhance uterine motility to evacuate its abnormal contents; induce estrus; increase mucus secretion which enhances the defense mechanisms, reduces progesterone levels, and increases estrogen level; and facilitate the action of antimicrobial agents (Olson 1996). Wide varieties of antimicrobial agents were used to control uterine infection (e.g., sulfonamides, tetracyclines, ß-lactamase, aminoglycosides, cephalosporins). Oxytetracycline (OTC) is one of antimicrobial agents approved by the FDA for use in lactating cows and thus specifically labeled for the treatment of endometritis caused by staphylococci and streptococci (Arrioja 2001). The environment of the postpartum endometritis such as low oxygen tension, antibiotics-degrading enzymes, mucopurulent discharge, and organic debris (Whitacre 1992) may diminish the efficacy of locally infused antibiotics. Therefore, it is important to select an antimicrobial agent which has the ability to overcome the most microbial load of the uterus such as OTC (Sheldon et al. 2004). OTC can be used as a systemic or intrauterine infusion which has an endometrium-restricted action (Kaczmarowski et al. 2004). Moreover, Cohen et al. (1995) reported extra merits and benefits for clinical uses of OTC like endometrium irritation, activating uterine defense mechanism, inflammatory cells, and promoting intrauterine leukocytes (PMNs) infiltration, in addition to the renewal of endometrium. Endometritis can be treated with intrauterine infusion as one of the most effective treatment protocols (Drillich et al. 2005). However, the results of treatment of clinical endometritis with OTC are still unable to eliminate bacterial endometritis completely. Thus, to combat microbial challenges and maximize antibiotics efficiency, nanoparticle oxytetracycline (OTC) 20% were experimented. This study aimed to evaluate the efficacy of the conventional OTC 5%, 20%, and OTC 20% nanoparticles infused intrauterine at fixed intervals (once per week for 3 consecutive weeks). The first dose of OTC was infused 3 days after injection with a single dose of PGF2α in order to evacuate the accumulated intrauterine discharges to combat postpartum clinical endometritis.

## Materials and methods

### Rectal and ultrasonographic diagnosis of clinical endometritis

Seventy-five postpartum lactating Holstein dairy cows making their 3rd lactation were used in this study. The cows belonged to a commercial dairy herd located in Abis area, Alexandria, Egypt. These cows were artificially inseminated more times without occurrence of pregnancy; they are classified as repeat breeder cases, whereas their estral mucous mixed with mucopurulent and purulent discharges were seen, as these cows have a sickness record of dystocia, birth help, and placental retention. Worth mentioning, all studied cows were raised under the same conditions of feeding, weather, and management. Also, all studied animals shared the same pathological conditions and also had the exact history of disease during the last calving. The genital tract was examined by rectal palpation as proposed by Grunert et al. (2005), and ovaries were checked for identifying ovarian structures and morphological alterations in the uterus and cervix. The uterine location, thickness, symmetry of horns, and consistency were also investigated concurrently with ultrasonographic scanning by SonoScape (model M12, China, with linear array trans-rectal probe 5-7.5 MHz) for assessment of ovarian structures, the diameter of the cervix, the uterus, and its contents as proposed by Meira et al. (2012). Cows with clinical endometritis were diagnosed ultrasonography which showed the presence of variable quantities of accumulated intrauterine fluid (Supplementary Figs. S1 and S2). Clinical endometritis was confirmed by intrauterine discharges with different degrees of echogenicity reflected by the density of mucopurulent or purulent discharges (Sheldon et al. 2006). Moreover, intrauterine accumulated fluids appear black (non-echogenic) when all waves pass through the medium without reflection. Hypoechoic appears when waves pass through the medium and many waves come back as referred to by Fissore et al. (1986). Indeed, a mixture of hypoechoic and hyperechoic appearance is obtained in the case of clinical endometritis.

### Treatment protocols and experimental design

Cows with clinical endometritis diagnosed by ultrasonography, serum concentration of cytokines, and acute phase proteins were assigned into three treated groups. All experimental cows received a single dose of PGF2α, 2 mL Estrumate® (500µg cloprostenol) (Vet Pharma, Friesory GmbH, Germany) (I/M) 3 days before starting intrauterine infusion of local antibiotics for enhancing myometrial contraction and evacuation of uterine contents to facilitate oxytetracycline action on the endometrium. Then, all cows were classified into three groups and infused with 3 intrauterine doses for 3 consecutive weeks (once per week at fixed intervals) as follows;

#### Oxytetracycline 5% group (OTCC5%)

Twenty-five cows received 20 mL oxytetracycline 5% as a local intrauterine infusion three times (once/week).

#### Oxytetracycline 20% group (OTCC20%)

Twenty-five cows were treated with 20 mL of oxytetracycline 20% as a local intrauterine infusion three times (once/week).

#### Oxytetracycline 20% nanoparticles group (OTC-NPs)

Twenty-five cows were treated with 10 mL oxytetracycline 20% nanoparticles (OTC-NPs) as a local intrauterine infusion three times (once/week).

**Fig.1:**
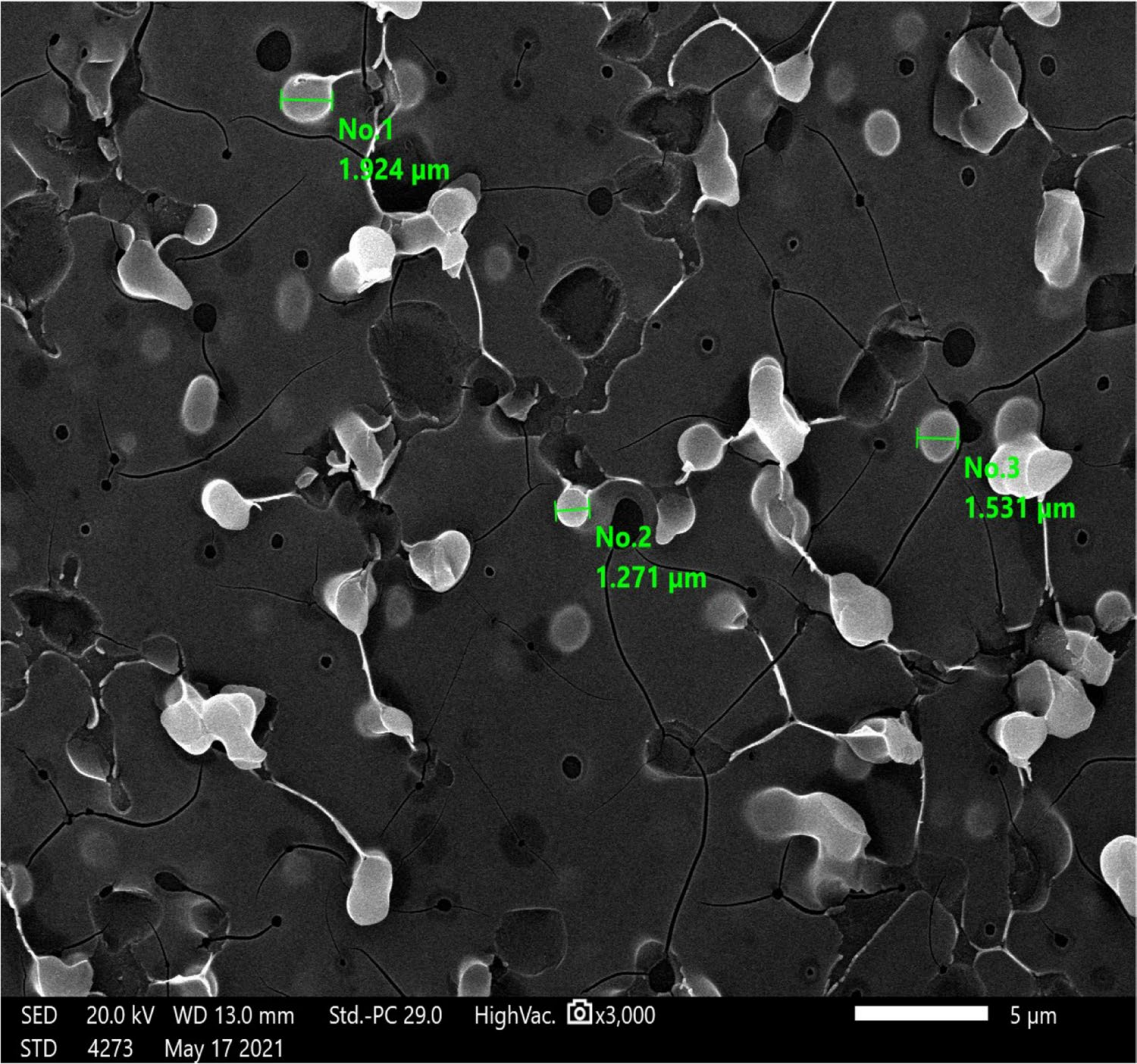
The size and shape of oxytetracycline molecules as appeared by electron microscope

**Fig. 2:**
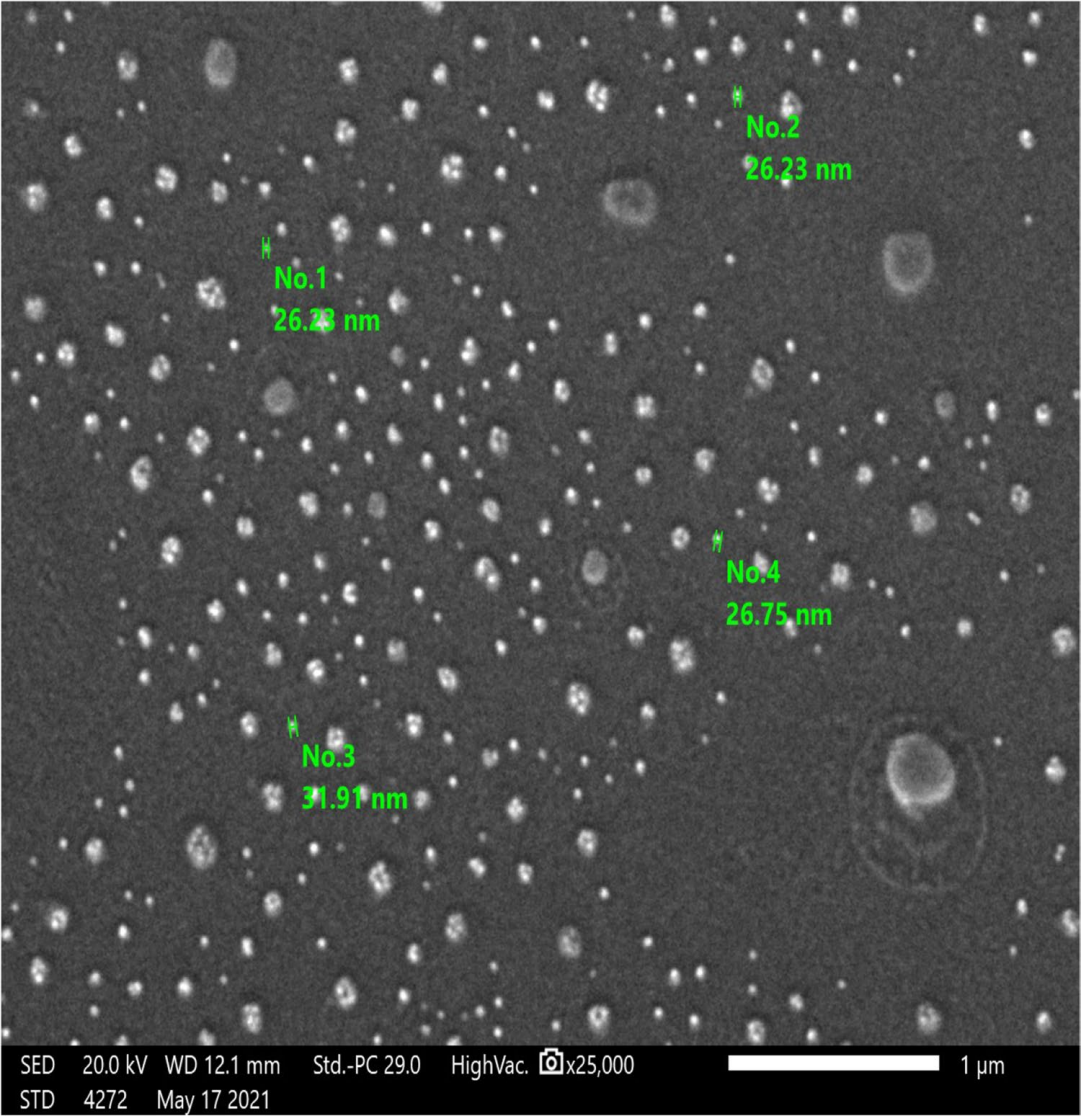
Oxytetracycline nanoparticles as appeared by scanning electron microscope

Oxytetracycline at different concentrations was used in this experiment, oxytetracycline 5% (Oxyvet-Pharma® 5%) injection, Pharma Swede-10th of Ramadan CityB3, Egypt, and oxytetracycline 20% (Oxytetra® 20% L.A. Kela N.V., Sint lenaartseweg 48, 2320 Hoogstraten, Belgium). Oxytetracycline 20% (Oxytetra® 20% L.A. Kela N.V., Sint lenaartseweg 48, 2320 Hoogstraten, Belgium) was used in this experiment, in order to synthesize oxytetracycline nanoparticles (OTC-NPs), whereas two litters of conventional oxytetracycline 20% L.A. (OTCC20%) were subjected to high microfluidic homogenization for 6 h at faculty of science, Alexandria University, according to the principles of Lammari et al. (2020). The size and shape of oxytetracycline molecules (OTCC20%) were scanned and measured before and after transformation into OTC-NPs by scanning emission microscope at the Faculty of Science, Alexandria University, as shown in Figs. 1 and 2. The scanning emission microscope photograph of OTCC20% particle’s shape almost appeared spherical, and their size ranged from 1.271 to 1.924 µm with an average of 1.575 µm equal to 1575 nm, and the shape appeared rounded attached to each other by thin filaments. Meanwhile, the photograph of OTC-NPs appeared spherical in shape with a thin filament properly on the side chain of OTCC, and their size ranged from 26.23 to 31.91 nm with an average of 27.7 nm, and the particle size appeared round without attachment by thin filaments. The OTC-NPs were found to be stable for more than 3 months without forming an aggregation or flocculation. The service morphology of OCT-NPs was analyzed at an accelerating voltage of 10.0 kV, mode, and detectors containing the second electron. The measurements revealed that molecules were reduced 56.6 times to obtain OTC nanoparticles. The conversion of OTC into nanoparticles reduces the particle size from 1.575 μm to < 27.78 nm.

### Blood sampling and pro-inflammatory cytokine assessment

Blood samples were collected from the jugular vein before starting the treatment (at the same day of first intrauterine infusion) and then three times on a weekly basis with second and third and one week after third intrauterine infusion for assessment of interleukins, acute phase proteins, C-reactive protein, total proteins, albumin, globulin, and albumin/globulin ratio to determine the rate of changes in the inflammatory indices. Serum total protein and albumin levels were measured calorimetrically using commercially available kits (Spectrum, Egypt). The serum globulins level was assessed as the difference between total protein and albumin. Also, the serum concentration of C-reactive protein** (**CRP) was determined using a rapid latex slide test commercial kit (Spectrum, Egypt). Besides, serum concentrations of interleukin-1 (IL-1), interleukin-6 (IL-6), and tumor necrosis factor-alpha (TNF-α) were detected using species-specific ELISA kits (Abcam, USA).

### Statistical analysis

Data of estrus interval and endometrial thickness were analyzed using GLM procedure of SAS Institute (2009) for testing the effect of treatments. Also, data of acute phase proteins (total proteins, albumin, globulin, albumin/globulin ratio, and C-reactive protein) and pro-inflammatory cytokines (IL-1, IL-6, TNF-α) were analyzed using GLM procedure for testing the treatment and time effects. Besides, the significant differences between means were tested using Duncan multiple range test (Duncan 1955). Also, chi-square analysis was used to test the effect of treatment on pregnancy rate at confidence interval of 95%.

## Results

The least square mean of treatment-estrus interval and endometrial thickness before and after treatment are presented in Table 1. It can be observed that the treatment-estrus intervals of the studied groups were different (*P*<0.001). The highest interval was for OTCC5% (17.70 day) followed by OTCC20% (13.59 day) then OTC-NPs (12.05 d). No differences were observed among groups for endometrial thickness (*P*=0.608) before treatment. In contrast, after treatment, OTC-NPs group showed the lowest endometrial thickness (5.53 mm), but OTCC5% cows had the highest thickness (15.40 mm). Furthermore, the least square mean of acute phase proteins (APP_s_), total proteins, albumin, globulin, and albumin/globulin ratio response of studied groups, are presented in Table 2. Treatment affects total protein concentrations (*P*<0.001). The highest serum concentrations of total proteins were observed for OTC-NPs cows in the three blood samples after applying the treatment protocol, but the lowest was obtained for OTCC5% in the same collections. The same trend was almost observed for albumin and globulin at all times after applying the treatment protocol. OTCC5% cows achieved the highest levels of total protein, albumin, and globulin in the first sampling only then all concentrations were declined with time to reach the lowest concentrations in the third time. Albumin/globulin ratio was the lowest at before treatment (*P*<0.05); then, no trend was observed among treatment groups in different samplings. Moreover, least square mean of pro-inflammatory cytokines, IL-1, IL-6, and TNF-α, and CRP response after application of treatments of clinical endometritis is presented in Table 3. The highest serum concentrations of pro-inflammatory cytokines were observed on OTCC5% cows first, second, and third collected blood samples, but the lowest was obtained for OTC-NPs cows at the same collections. Moreover, all studied pro-inflammatory cytokines decreased with time for OTC-NPs cows and the smallest values were observed on the third sampling being; 10.11, 99, and, 46 for IL-1, IL-6, and TNF-α response, respectively. Also, CRP decreased with time in the two OTC groups. CRP had the lowest values for the three blood samples after the application of treatments for OTC-NPs cows. The smallest value of CRP (1.01) was achieved on the third sampling for OTC-NPs cows. The reproductive performance of OTCC5%, OTCC20%, and OTC-NPs treatment groups is presented in Table 4. No differences were observed among groups for the number of services per conception. Cows of OTC-NPs achieved the largest conception and pregnancy rates (77, 72 %) followed by the OTCC20% group (63, 63 %), but the OTCC5% had the smallest (50, 10 %, respectively).

**Table 1:** Least square means of treatment estrus interval and endometrial thickness for oxytetracycline 5% (OTCC 5%), oxytetracycline 20% (OTCC 20%), and oxytetracycline 20% nanoparticles (OTC-NPs) after application to clinical endometritis diseased cows

Traits	Treatment			SEM^1^	*p* value
	OTCC 5%	OTCC 20%	OTC-NPs		
Treatment estrus interval, day	17.70^a^	13.59^b^	12.05^c^	0.316	<0.001
Endometrial thickness, mm:					
Before treatment	14.78	14.04	14.23	0.226	0.608
After treatment	15.40^a^	7.27^b^	5.53^c^	0.358	<0.001

^1^*SEM*, standard error of mean

^a–^^c^Means with different letters within the same row are differ (*P* < 0.05)

**Table 2:** Least square means of acute phase proteins: total proteins, albumin, globulin, and albumin/globulin ratio for oxytetracycline 5% (OTCC 5%), oxytetracycline 20% (OTCC 20%), and oxytetracycline 20% nanoparticles (OTC -NPs) after application to clinical endometritis diseased cows

Traits^*^	Treatment			SEM	*P*-Value
	OTCC 5%	OTCC 20%	OTC-NPs		
Total protein, g/dL					
Pre	7.10^Aa^	6.47^b^	6.37^b^	0.061	<0.001
First	5.86^Bb^	6.22^a^	6.44^a^	0.056	<0.001
Second	4.58^Cb^	6.18^a^	6.70^a^	0.135	<0.001
Third	3.44^Db^	6.06^a^	6.62^a^	0.205	<0.001
Albumin, g/dL					
Pre	2.14^B^	2.13	2.27	0.052	0.695
First	2.64^Ac^	3.11^b^	3.32^a^	0.045	<0.001
Second	2.13^Ba^	3.10^a^	3.03^b^	0.081	<0.001
Third	1.70^Cb^	3.04^a^	3.34^a^	0.111	<0.001
Globulin, g/dL					
Pre	4.91^Aa^	4.29^b^	4.10^c^	0.033	<0.001
First	3.20^B^	3.09	3.10	0.029	0.407
Second	2.38^Cb^	3.08^a^	3.61^a^	0.056	<0.001
Third	1.78^Dc^	3.00^b^	3.25^a^	0.102	<0.001
Albumin/globulin ratio					
Pre	56.85^C^	64.88^B^	63.13^C^	1.605	0.055
First	82.23^Bb^	98.25^Aa^	101.16^Aa^	1.602	<0.001
Second	88.56^ABb^	100.71^Aa^	94.76^Aab^	1.912	0.011
Third	94.74^A^	101.90^A^	88.36^B^	2.138	0.099

^1^*SEM*, standard error of mean

^a–^^c^Means with different letters within the same row are differ (*P* < 0.05)

^A–^^D^Means with different letters within the same column are differ (*P* < 0.05)

^*^Pre: blood samples were collected at the same day of first intrauterine infusion. First: blood samples were collected 1 week after first OTCC intrauterine infusion. Second: blood samples were collected 1 week after second OTCC intrauterine infusion. Third: blood samples were collected 1 week after third OTCC infusion

**Table 3:** Least square means of pro-inflammatory cytokines; interleukin-1 (IL-1), interleukin-6 (IL-6), tumor necrosis factor-alpha (TNF-α), and C-reactive protein (CRP) for oxytetracycline 5% (OTCC 5%), oxytetracycline 20% (OTCC 20%), and oxytetracycline 20% nanoparticles (OTC-NPs) after application to clinical endometritis diseased cows

Traits^*^	Treatment			SEM	*p* value
	OTCC 5%	OTCC 20%	OTC-NPs		
IL-1, pg/mL					
pre	30.90^b^	31.89^ab^	34.13^Aa^	0.487	0.031
first	32.13^a^	29.63^b^	21.92^Bc^	0.590	<0.001
second	33.21^a^	27.08^b^	14.85^Cc^	0.937	<0.001
third	35.56^a^	25.45^b^	10.11^Dc^	1.158	<0.001
IL-6, pg/mL					
pre	310^b^	333^a^	314^Ab^	3.288	0.011
first	327^a^	315^a^	213^Bb^	7.131	<0.001
second	338^a^	308^b^	137^Cc^	10.986	<0.001
third	319^a^	300^a^	99^Db^	12.366	<0.001
TNF, pg/mL					
pre	239	256	240^A^	3.368	0.064
first	260^a^	236^b^	138^Bc^	6.724	<0.001
second	240^a^	206^b^	79^Cc^	8.364	<0.001
third	266^a^	183^b^	46^Dc^	9.837	<0.001
CRP, mg/L					
pre	7.27	7.10^A^	7.49^A^	0.129	0.374
first	7.45^a^	6.12^Bb^	4.20^Bc^	0.184	<0.001
second	7.13^a^	5.17^Cb^	2.19^Cc^	0.233	<0.001
third	8.06^a^	4.40^Db^	1.01^Dc^	0.279	<0.001

^1^*SEM*, standard error of mean

^a–^^c^Means with different letters within the same row are differ (*P* < 0.05)

^A–^^D^Means with different letters within the same column are differ (*P* < 0.05)

^*^Pre: blood samples were collected at the same day of first intrauterine infusion. First: blood samples were collected 1 week after first OTCC intrauterine infusion. Second: blood samples were collected 1 week after second OTCC intrauterine infusion. Third: blood samples were collected 1 week after third OTCC infusion

**Table 4:** Reproductive performance traits of cows after treatment of clinical endometritis for oxytetracycline 5% (OTCC 5%), oxytetracycline 20% (OTCC 20%), and oxytetracycline 20% nanoparticles (OTC-NPs) treatments

Traits	Treatment			SEM	*P* value
	OTCC 5%	OTCC 20%	OTC-NPs		
Number of services per conception, no	2.70	2.41	2.26	0.132	0.235
Conception rate, %	50^b^	63^ab^	77^a^	0.090	0.012
Pregnancy rate, %	10^b^	63^a^	72^a^	0.082	0.001

^1^*SEM*, standard error of mean

^a–^^b^Means with different letters within the same row are differ (*P* < 0.05)

## Discussion

Different attempts have been made to combat postpartum clinical endometritis which may destroy the future fertility of dairy cows. A wide range of antibiotics were applied systemically or by intrauterine infusion with variable efficacy. Oxytetracycline acts on a wide range of microbes in addition to its effective action anaerobically postpartum which made it the drug of choice to treat clinical endometritis by intrauterine infusion (Kaczmarowski et al. 2004). In contrast, Cohen et al. (1995) reported that OTC couldn’t penetrate uterine wall effectively and didn’t eliminate uterine infection caused by *T. pyogenes*, in addition, its irritation to the endometrium mucosa. Makki et al. (2016) reported an improvement in reproductive status in the OTCC group compared to the hyper-immune serum group for treatment of clinical endometritis. However, a need for maximizing the efficiency of oxytetracycline in treating uterine inflammation necessitated developing modern ways of utilization and turning it into nanoparticles like the present study. In the current study, the diagnosis of clinical endometritis was performed via assessment of the concentration of pro-inflammatory cytokines (IL-1, IL-6, and TNF-α) and APPs (CRP, total proteins, albumin, and globulin) in the serum of cows with clinical endometritis and recorded higher serum concentration of pro-inflammatory cytokines which was associated with clinical endometritis. This increase may be attributed to the increased activity of immunocompetent cells in the uterus. This action doesn’t emerge in healthy uterine tissue. Indeed, the majority of uterine microbes were eliminated early after up to 21 days after calving in normal uterine involution. In contrast, delaying postpartum uterine clearance and involution predispose dairy cows to clinical endometritis (Sheldon et al. 2006). Therefore, the local uterine defense mechanism is then activated according to the number and pathogenicity of microbes. Furthermore, clinical endometritis in the present study was confirmed by ultrasonography and showed a significant increase of intrauterine accumulated discharges with variable degrees of echogenicity according to the nature of discharges. Moreover, an increase in uterine wall thickness with different degrees of echotexture was detected by the ultrasound images (supplementary Figs. S1 and S2). The higher concentration of cytokines such as TNF-α, IL-1β, and IL-6 in the serum of cows with clinical endometritis in the present study was consistent with those obtained by Turner et al. (2012) and Kasimanickam et al. (2013) when compared to the healthy uterus and also was in agreement with Kasimanickam et al. (2005) and Brodzki et al. (2014a, b a, b); the results on the basis of the presence of pathogens activate the release of large numbers of uterine neutrophils displaying an increase of pro-inflammatory cytokines mainly IL-6, TNF-α, and IL-1. Moreover, IL-6 has a fundamental role in the uterine intracellular communication resulting in accurate diagnosis of clinical endometritis (Loyi et al. 2013). Contrary to the above-mentioned, Kim et al. (2014) and Ishikawa et al. (2004) didn’t observe any difference between the concentration of cytokines of healthy and diseased uterus. Also, the present study revealed an increase in the concentration of APPs and CRP, which was in agreement with Kaya et al. (2016) who reported that pro-inflammatory cytokines are the primary initiators of APPs such as CRP, PMNs %, haptoglobin, and serum amyloid A and regulated by secretion of IL-1, IL-6, and TNF-α. Hence, the high serum levels of TNF-α, interleukins, and CRP could introduce a logical alternative to diagnose clinical endometritis. Our results indicated that a high concentration of serum cytokines together with the presence of intrauterine accumulated fluids with different degrees of echogenicity confirmed the diagnosis of clinical endometritis and matched with Sheldon et al. (2009) and LeBlanc et al. (2002) who declared that a severe form of endometritis induced foul purulent discharges as early as 20 to 33 days postpartum, like purulent discharges in this study (supplementary Fig. S4). It is noteworthy that the positive effect of PGF2α treatment on fertility in the present study is attributed to the improvement in uterine tonicity, intrauterine discharges evacuation, and improve the uterine environment that gave a real chance for the action of OTCC20% (20 mL) or OTC-NPs (10 mL) infused intrauterine which were in accordance with results of Kasimanickam et al. (2005) who explained that PGF2α didn’t affect the prevalence of endometritis but acts mainly by inducing estrus in cows having corpus luteum as well as increased physical clearance of the uterine media and its defense mechanism. The present results showed a fundamental gradual decrease in the level of pro-inflammatory cytokines in OTC-NPs then OTCC20% and OTCC5% groups throughout the progress of treatment period, especially in the third blood sample collection. The elevated concentration of IL-6, as well as other interleukins, act as an initiator for APPs release and an indicator for the presence of uterine microbial challenges which were similar with results of Loyi et al. (2013) and Brodzki et al. (2014a, b and 2015). Similar to our results, Ahmad et al. (2004) found high levels of total proteins in the serum of cows suffering from endometritis and low concentration in cyclic cows. Regarding serum albumin and globulin concentration in the current study, they could be used as an indicator of clinical endometritis when the level of albumin decreased and globulins increased and that were matched with the results of Whitaker et al. (1999). It is noteworthy in this study that globulin was released due to an inflammatory response associated with infection but decreased after the application of OTCC20% and OTC-NPs treatment protocols which coincided with the results of Makki et al. (2016). An interpretation was offered by Green et al. (2009) and Burke et al. (2010), whereas cows with clinical endometritis had lower concentrations of serum albumin due to liver dysfunctions. Meanwhile, increasing albumin concentration back to the normal level is a sign of a subsiding uterine inflammatory process, reduction of the immune response, decreased protein catabolism, improvement of liver functions, and further the disappearance of intrauterine discharges. It was evident that clinical endometritis caused endometrium enlargement and increased uterine wall thickness depending on the severity and duration of the infection (Leblanc et al. 2002). This was matched with echotexture and echogenicity examination of uterine wall and contents monitored with ultrasonography (supplementary Figs. S1 and S2). Meanwhile, the reduction in the uterine wall thickness, the disappearance of intrauterine discharge, and the improvement of the general status of the uterus after application of OTC-NPs and OTCC20% (supplementary Figs. S3 and S4) suggested the diminished inflammatory reaction due to the cow recovery from clinical endometritis. In addition, an improvement in the reproductive performance traits for the number of services per conception, conception rate, and pregnancy rate was observed. It was obvious that all current studied parameters had a tendency to decrease after administration of OTC-NPs and also conception and pregnancy rates increased. Besides, the treatment-estrus interval was the shortest (12.05 days) after OTC-NPs while was the longest (17.7 days) for OTCC5%. This may confirm the improvement in the reproductive status of cows after applying treatment protocols. The overall visible improvement in reproductive performance and uterine conditions was much related to the conversion of OTCC20% to OTC-NPs. The nano formula reduced the molecular size of OTCC20% 56.6 times (supplementary Figs. 2 and 4). This allowed OTC-NPs to possess new physical and chemical features among which effective therapeutic properties that realize more bioavailability of the substance to the uterine tissues which facilitate the spread of the drug to cover all target cells or tissue surface area of the endometrium using less dosage (10 mL instead of 20 mL). In addition, it had the ability to work in the presence of mucopurulent secretions that would reduce the action of OTCC20%. Also, OTC-NPs had a vital role in reducing microbial resistance.

This was fully coordinated with the other items of the current study like concentration of pro-inflammatory cytokines, acute phase proteins, size of endometrium thickness, enlargement of uterine walls, and disappearance of intrauterine discharges detected by ultrasonography examination that confirmed the efficiency of OTC-NPs local infusion in treating clinical endometritis.

## Conclusion

Conversion of OTCC20% to OTC-NPs increased the chance of dairy cows’ recovery from clinical endometritis. Converted OTC-NPs possess more effective properties for the treatment of clinical endometritis than OTCC20%. This improvement may be due to attaining new physical, chemical, and therapeutic efficacy than OTCC20%. Diagnosis, the severity of infection, disease withdrawal, and recovery can be detected by measuring the serum concentration of pro-inflammatory cytokines and acute phase protein concurrently with ultrasonographic monitoring of uterine echogenicity and echotextures in addition to the performance of reproductive functions. The OTC-NPs in the present study revealed a visible improvement in lowering serum inflammatory cytokines, acute phase proteins, and endometrium thickness in addition to improving reproductive performance. The reduced OTC-NPs particles permit high bio-availability and better spread that cover the target tissues using low dosage to kill the pathogen or make it vulnerable.

## Supplementary Information

Below is the link to the electronic supplementary material.

Supplementary file1 (PDF 893 KB)

## Data Availability

Not applicable.
