# Supplementary material for: Efficiency of conventional and nanoparticle oxytetracycline in treatment of clinical endometritis in postpartum dairy cows
Source: Trop Anim Health Prod. 2023 Mar 17;55(2):118. doi: 10.1007/s11250-023-03536-0 (PMC10023628; doi:10.1007/s11250-023-03536-0)
Supplement: Supplementary file 1 — Supplementary file1 (PDF 893 KB) [file 11250_2023_3536_MOESM1_ESM.pdf]

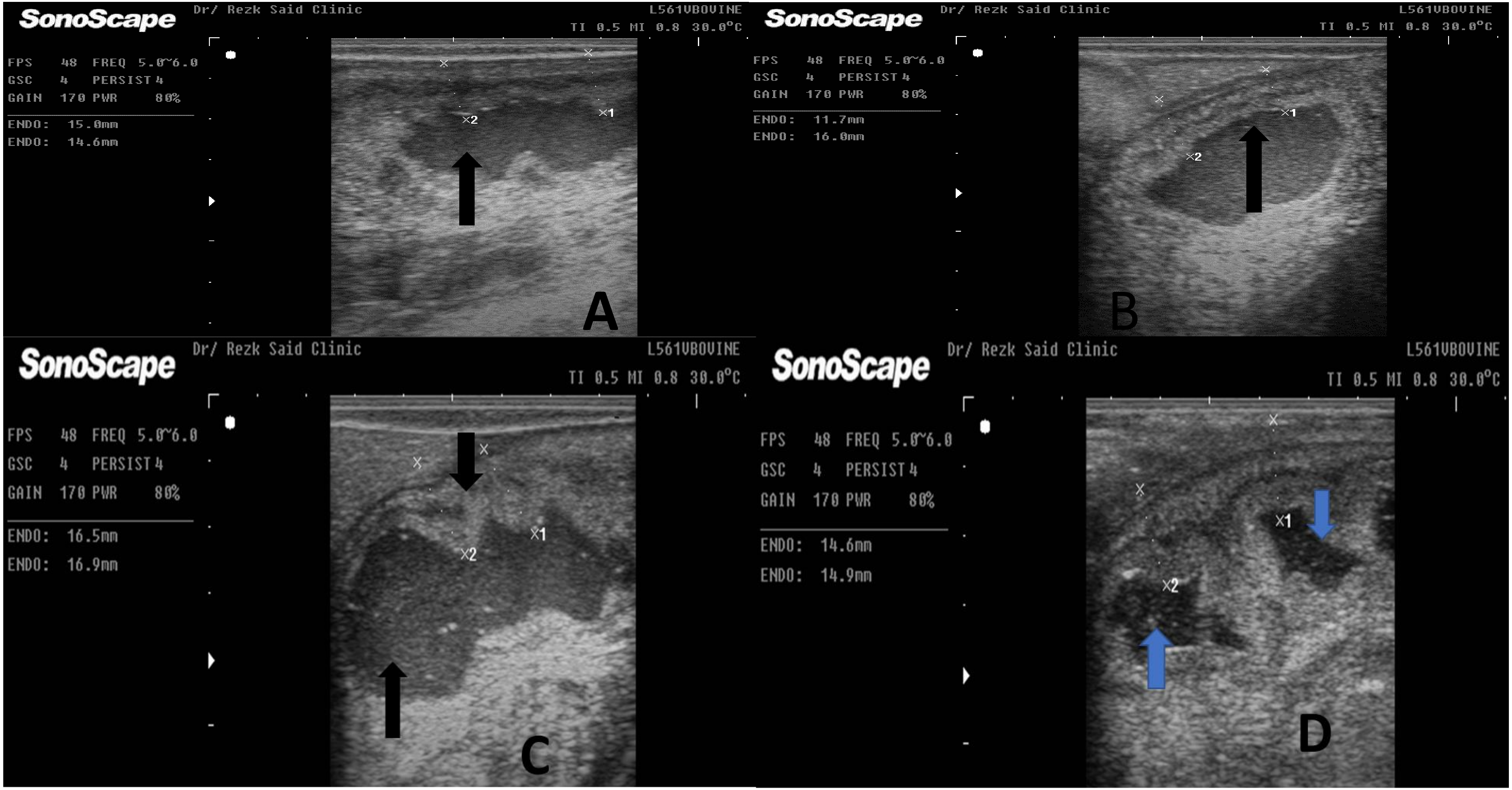

Fig S1. Ultrasonography monitoring intrauterine discharges accumulated with different degree of echogenicity and subsequent thickening of uterine wall with variable degree of echotexture in postpartum cows with clinical endometritis .

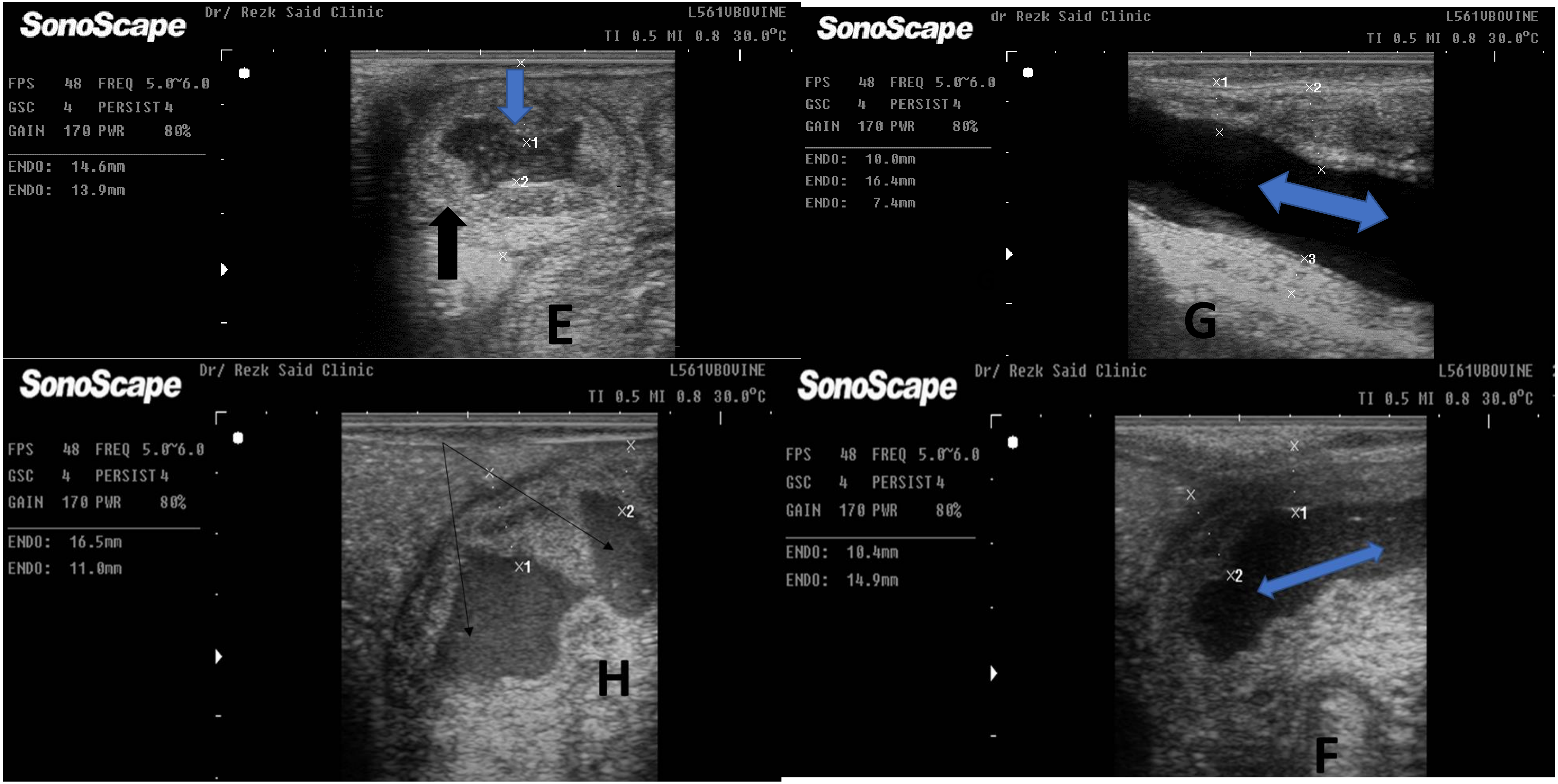

Fig S2. H &E a cross section ultrasonographic image of uterine horns containing uterine discharges, meanwhile, G &F are longitudinal section of ultrasonographic image over uterine body containing intrauterine discharges with different degree of echogenicity and echotexture in postpartum cows with clinical endometritis.

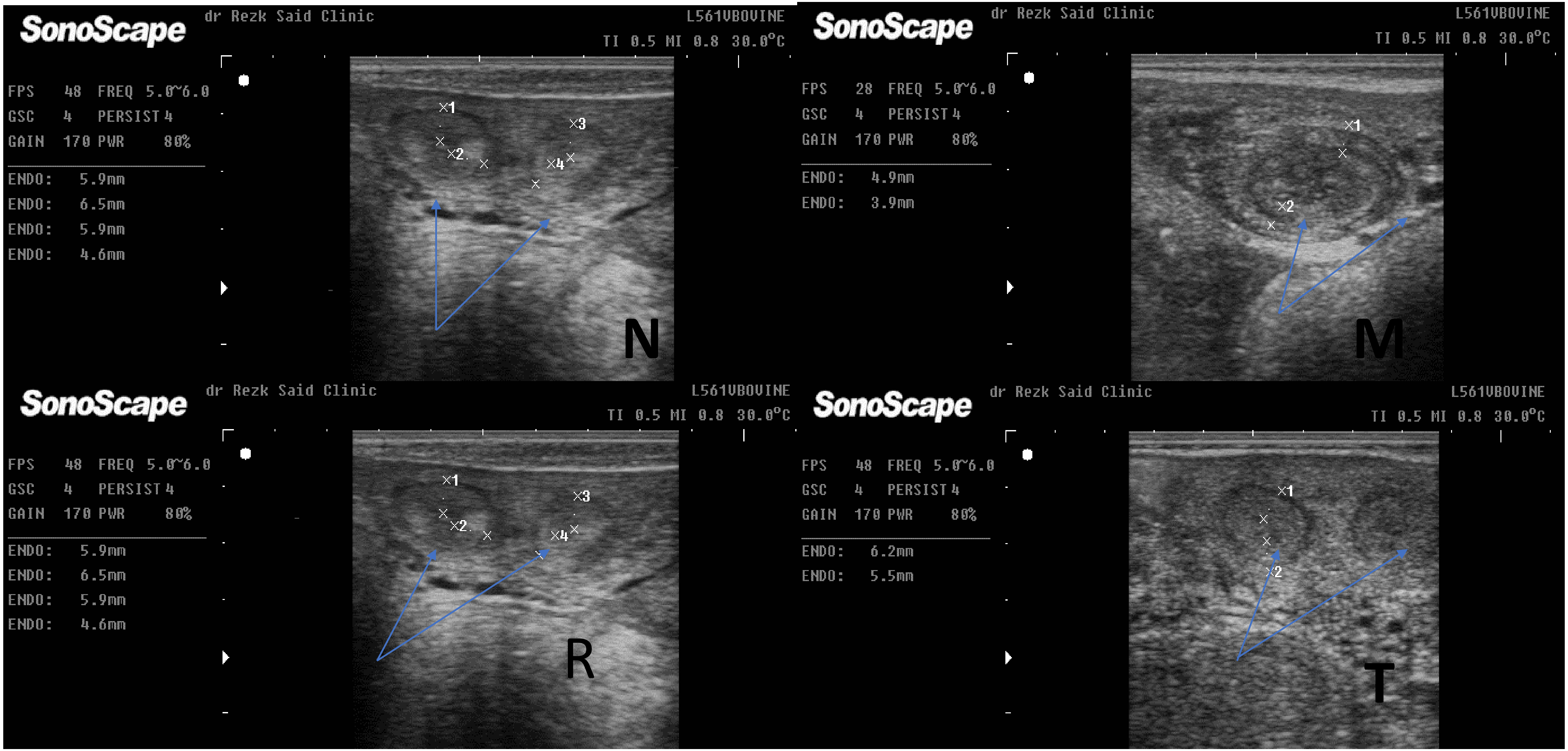

Fig S3. Ultrasonographic images show a cross section of uterine horns after application of treatment protocols and disappearance of intrauterine discharges as well as reduction of uterine wall thickening to half its size before treatment in clinical endometritis cows.

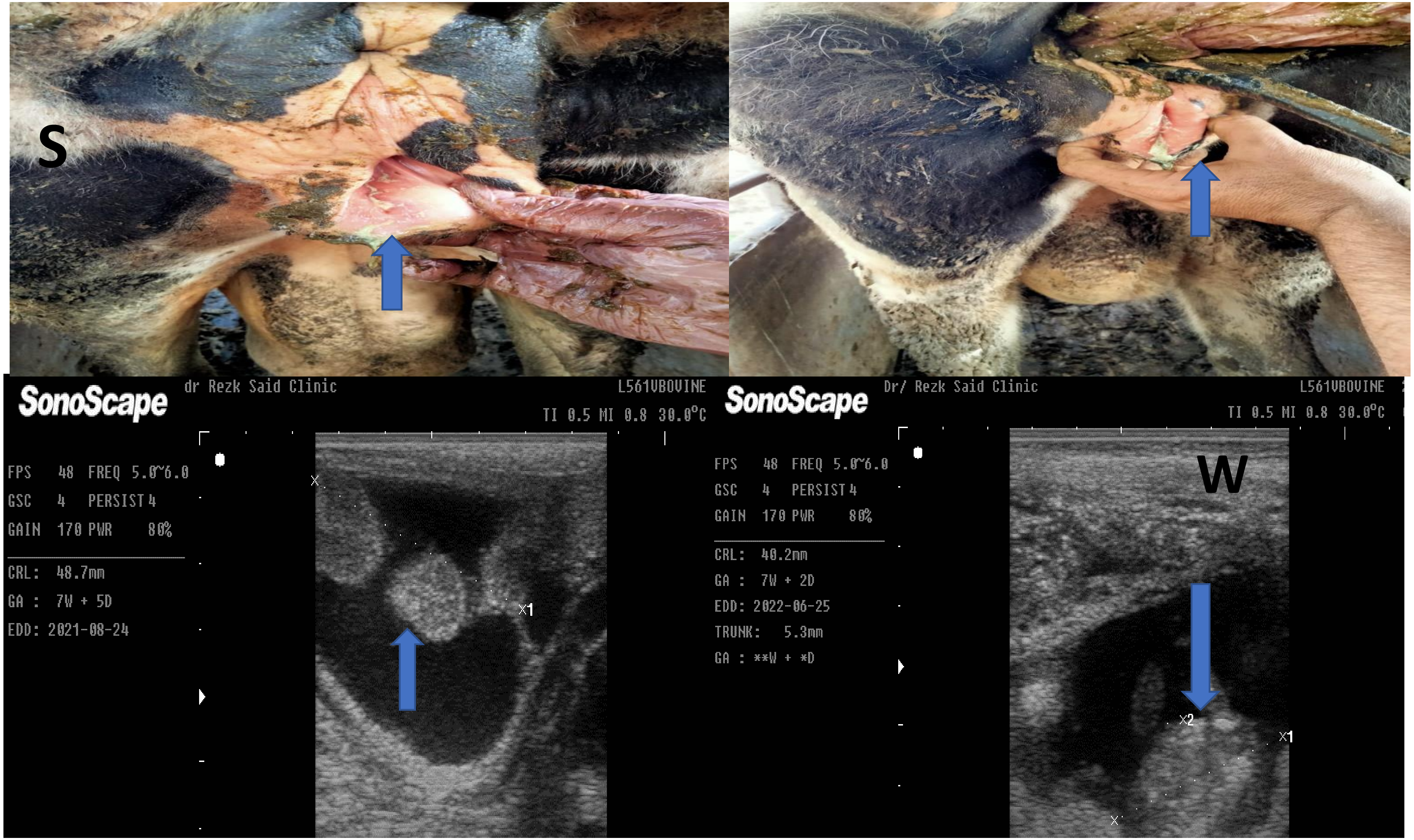

Fig S4. S, shows mucopurulent and purulent discharges on vulva lips of clinical endometritis cows. W, show a pregnant uterus containing amniotic vesicles surrounded by foetal fluids.
